# The hidden breast lesions: A case report of bilateral breast cancer

**DOI:** 10.1002/jcu.23117

**Published:** 2021-12-25

**Authors:** Chaochao Dai, Lingyun Bao, Yanjuan Tan, Chenxiang Jiang

**Affiliations:** ^1^ Department of Ultrasonography Affiliated Hangzhou First People's Hosptital, Zhejiang University School of Medicine Hangzhou China

**Keywords:** breast, magnetic resonance imaging, mammography, ultrasound

## Abstract

Bilateral breast cancer (BBC) is rare and is associated with an unfavorable prognosis. Consequently it is crucial to improve diagnostic performance of breast cancer in the clinical setting. We report a case of BBC in a 66‐year‐old woman and describe the imaging findings, including mammography, hand‐held ultrasound, automated breast ultrasound, anatomical intelligence for breast ultrasound (AI‐breast), and magnetic resonance imaging. Only AI‐breast ultrasound successfully located the two tumors, while other imaging examinations failed to detect the tumor in the right breast.

## Graphical abstract

AI Breast successfully located the two tumors, while in the automated breast ultrasound scanning, HHUS scanning and MRI examination, the tumor in the right breast was miss‐detected. we evaluated the efficiency of AI Breast in breast lesion scanning and detection was investigated.

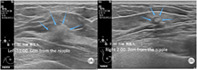

## 1 INTRODUCTION

The occurrence of bilateral breast cancer (BBC) is increasing nowadays with a reported incidence ranging between 1.4% and 12%.^1^, ^2^ However, in recent literature, patients with BBC showed a significantly worse distant relapse‐free survival (RFS) as compared to those with unilateral breast cancer (UBC), as distant metastases were frequently reported in patients with BBC.^2^, ^3^ Early detection and treatment of BBC is important to improve the overall prognosis of patients with breast cancer.

## 2 CASE REPORT

A 66‐year‐old female patient with a palpable mass in the left breast found by self‐examination came to our hospital for further investigation. Mammographic findings from another hospital included focal asymmetric density in the upper inner quadrant of the left breast. The patient had annual breast cancer screening by hand‐held ultrasound (HHUS), but no breast lesions were found in the past 3 years. There was no family history of breast cancer. The patient had a history of total hysterectomy for endometrioid adenocarcinoma, and partial thyroidectomy for thyroid adenoma and nodular goiter. The values of CA125 and CEA were normal.

**FIGURE 1:**
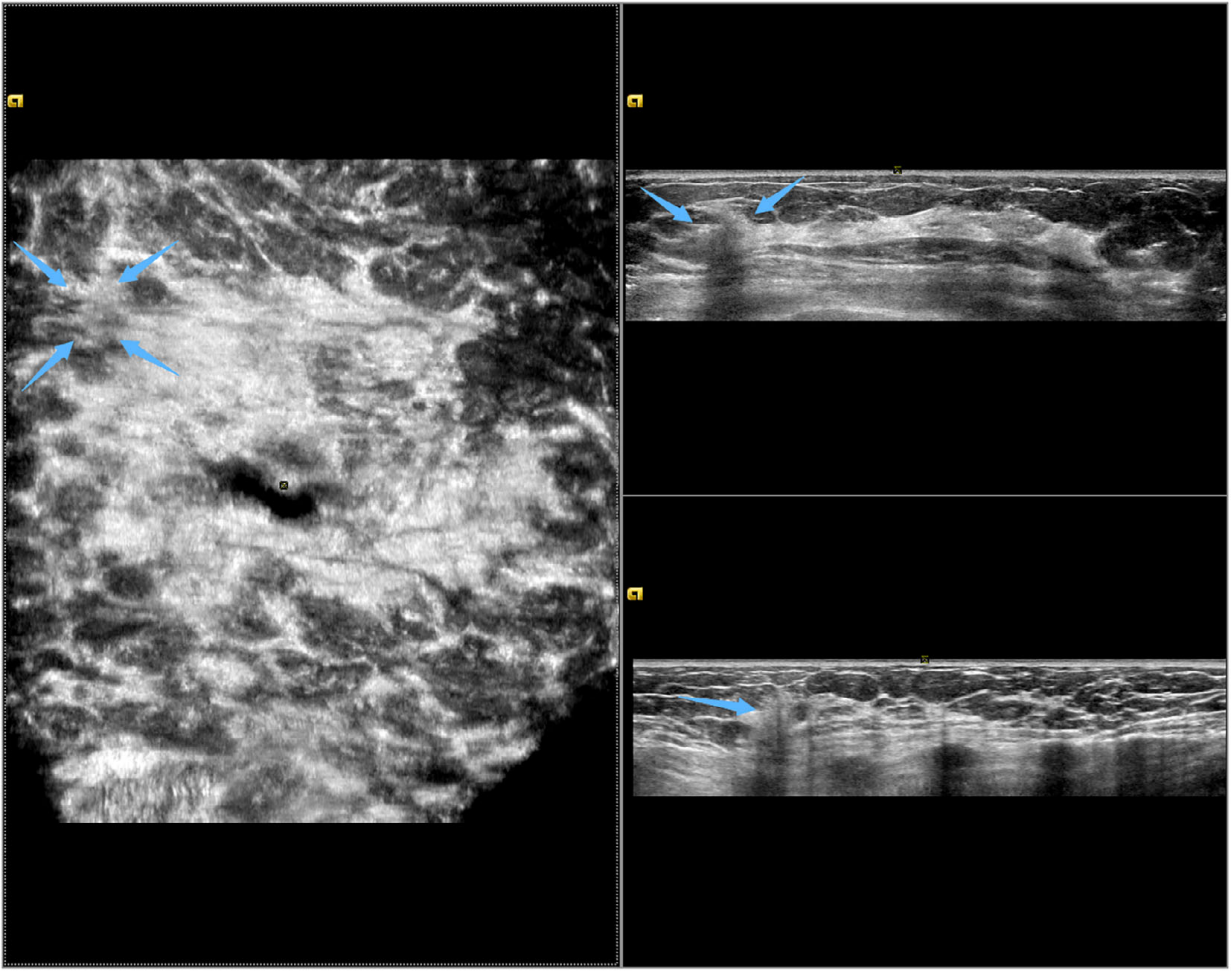
The left breast lesion in automated breast ultrasound (US). The left anteroposterior view reveals a non‐mass‐like breast lesions in the 10‐ to 11‐o'clock position 7 cm away from the nipple (blue arrows)

**FIGURE 2:**
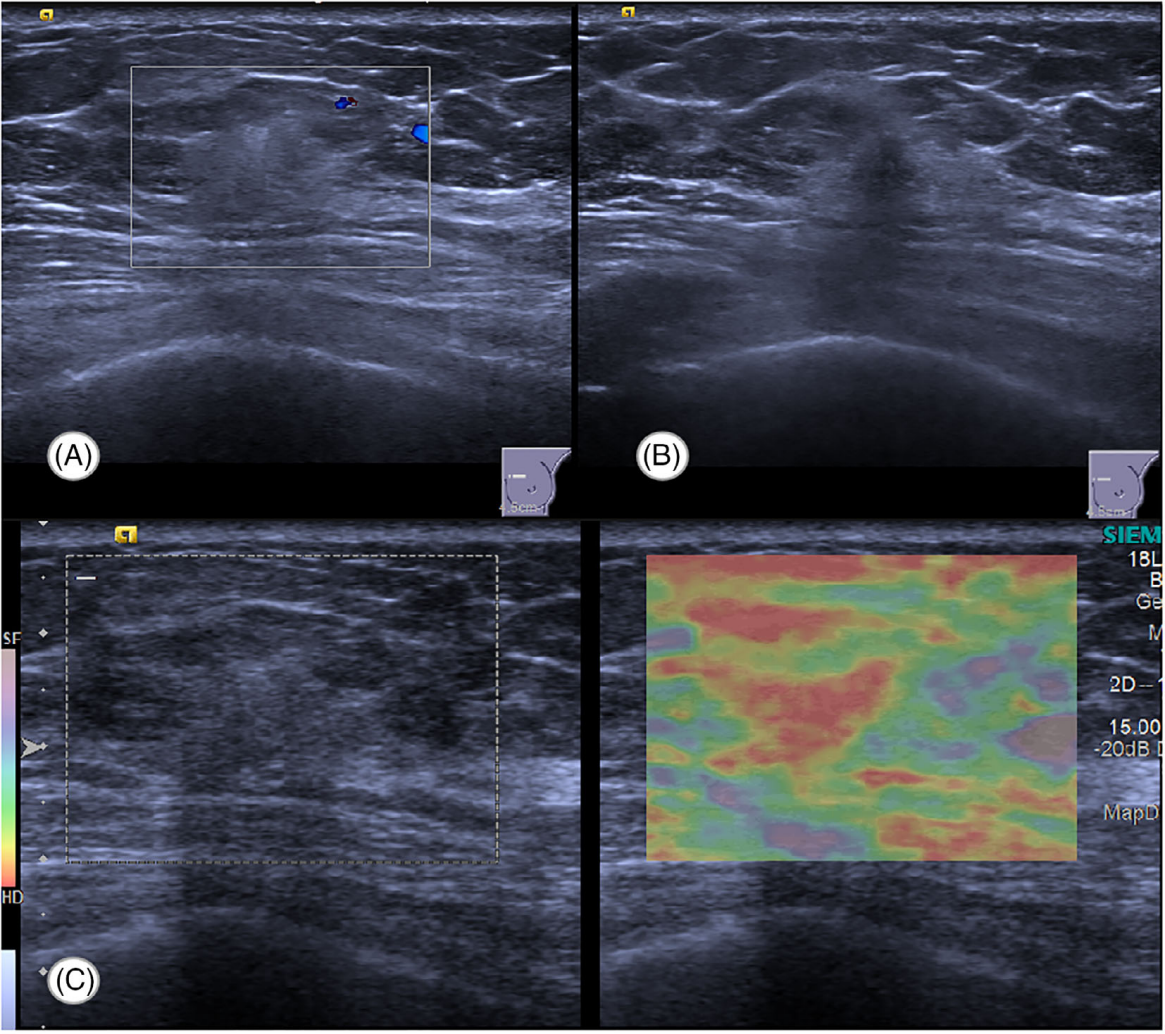
Ultrasonic finding of the left lesion by hand‐held ultrasound (HHUS). (A) Color Doppler sonogram shows no internal vascularity within the lesion. (B) Gray‐scale transverse view of the left breast demonstrates a heterogeneous hypoechoic lesion in the 10 o'clock position. (C) Elastography revealed a Tsukuba stiffness score of 3

Physical examination identified a less mobile, hard mass with unclear border measuring 15 mm in diameter in the upper inner quadrant of the left breast. The examinations of the right breast and axillary lymph nodes were unremarkable. With these clinical examination findings, there was suspicious indication for surgery. The combination of HHUS and automated breast ultrasound scanning was recommended for further evaluation. Automated breast ultrasound scanning was used to acquire datasets for two‐view scans: the lateral and the anteroposterior (AP) view. By reviewing the acquired scanning datasets, an ill‐defined heterogeneous hypoechoic zone (18 × 13 × 18 mm) with posterior acoustic shadowing was observed in the 10‐ to 11‐o'clock position, 7 cm away from the nipple of the left breast. There were no microcalcifications, architectural distortion or mass within the territory (Figure 1). The convergence sign was not observed on the coronal plane. No lesion was detected in the right breast. By conventional HHUS, color Doppler was performed on the hypoechoic lesion in the left breast and no internal color flow signals were detected. Elastography resulted in a Tsukuba stiffness score of 3 (Figure 2). According to the Ultrasound Breast Imaging Reporting and Data System (BIRADS), the lesion was classified as BIRADS 4A.

**FIGURE 3:**
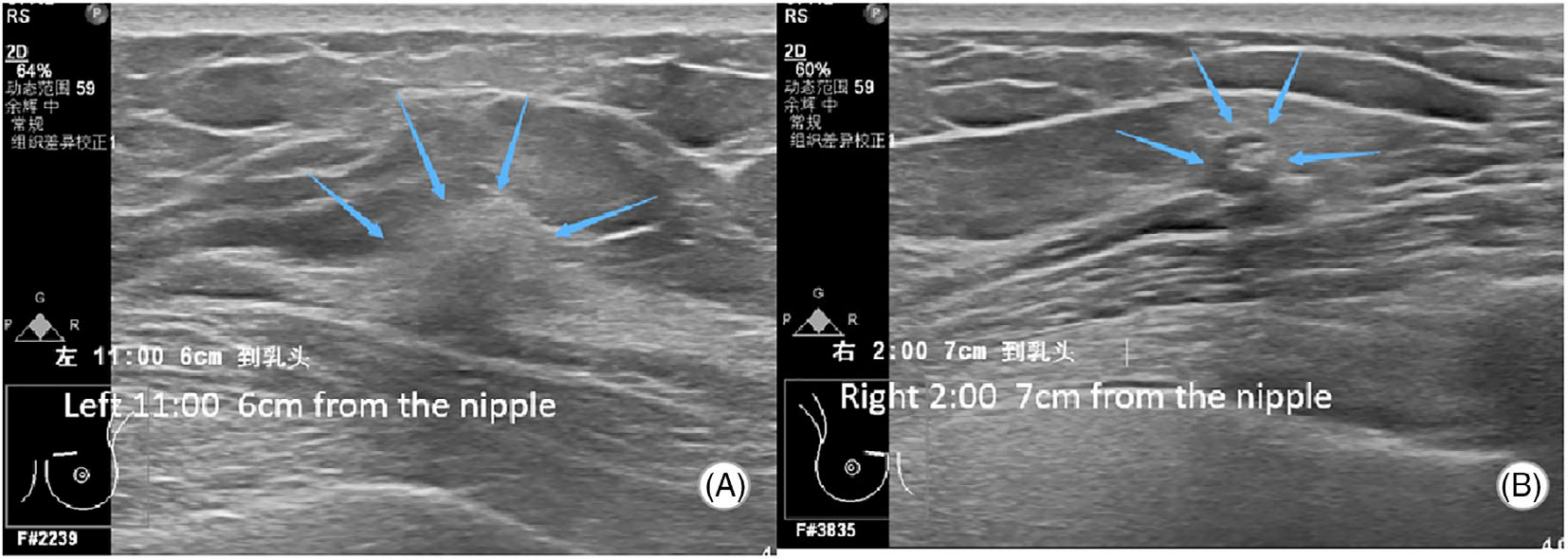
Ultrasonic findings of the bilateral breast lesions by AI‐breast US. (A) A heterogeneous hypoechoic lesion detected in the left breast in the 10‐ to 11‐o'clock position 6 cm away from the nipple. (B) A nodule with calcifications within the mass detected in the right breast in the 2‐o'clock position 7 cm away from the nipple

The US Department was running a prospective clinical trial to study the efficiency of AI‐breast and HHUS in breast lesion screening. This patient met the inclusion criteria and agreed to participate in the trial. Therefore, AI‐breast scanning was additionally performed on the patient to detect breast lesions. The general radiologist interpreting the scan images and a technician for AI‐breast were both blinded to the results of other US examinations. In AI‐breast scanning, besides the same lesion detected in the left breast (Figure 3A), another lesion was detected in the right breast (Figure 3B). The lesion located in the 2‐o'clock position 7 cm away from the nipple of the right breast was irregular and indistinct with posterior acoustic shadowing. Calcifications observed within the mass were mostly macro‐calcifications.

**FIGURE 4:**
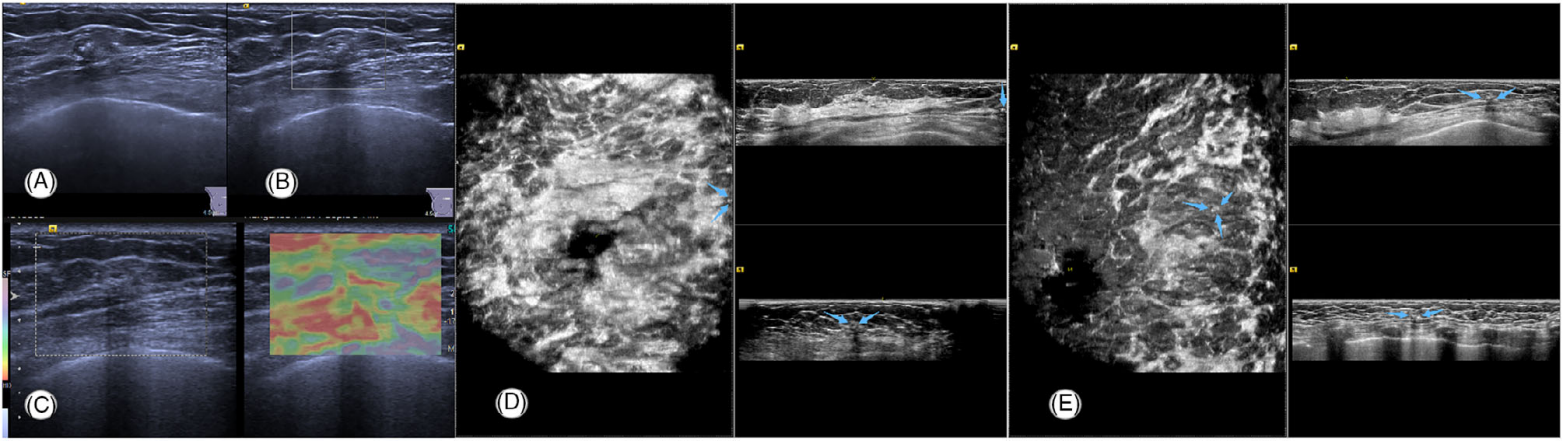
Ultrasonic finding of the right lesion by HHUS and automated breast US. (A) An irregular and indistinct hypoechoic nodule with calcifications detected in the 2‐o'clock position of the fat layer. (B) Color Doppler US reveals no internal color flow signals. (C) Elastography revealed a Tsukuba stiffness score of 2. (D, E) Anteroposterior (AP) view and medial (MED) view by AI‐breast US demonstrates a hypoechoic nodule 7 cm away from the nipple of the right breast (blue arrows), but only part of the lesion is shown on AP view

Upon reviewing the data of the AP view of the right breast, a nodule which was not whole was observed on the edge of the 2 o'clock position. The patient was recalled for second‐look US on the right breast and the medial view was added. With reference to the position of the lesion located by AI‐breast scanning, a hypoechoic nodule (8 × 5 × 6 mm) in the right breast with calcifications in the mass was detected (Figure 4), which was irregular, indistinct and parallel to the chest wall. Similar ultrasonic findings were observed which were consistent with the findings of AI‐breast ultrasound. No vessel was detected by color Doppler scanning. The lesion in the right breast was finally classified as BIRADS 4A.

**FIGURE 5:**
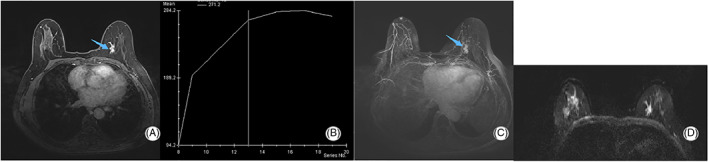
Findings of MRI examination. (A) An area of non‐mass enhancement is detected. (B) A type II enhancement curve is observed. (C) Maximum signal intensity projection image indicates enlarged blood vessels surrounding the lesion in the left breast. (D) Diffusion‐weighted imaging examination reveals mild diffusion limitation

To further confirm the surgical plan, a recommendation was made for contrast‐enhanced magnetic resonance imaging (MRI) examination. MRI revealed an irregular zone (22 × 12 × 12 mm) with focal heterogeneous non‐mass enhancement in the left breast (Figure 5A). The time–signal intensity curve (TIC) showed a type II enhancement curve (plateau pattern, Figure 5B). On the maximum signal intensity projection image, enlarged blood vessels were observed surrounding the lesion in the left breast and mild diffusion limitation was found on the diffusion‐weighted image (Figure 5C and D). Based on all the MRI findings, the lesion in the left breast was classified as BIRADS 4. No characteristics suggestive of malignancy were observed in the right breast.

**FIGURE 6:**
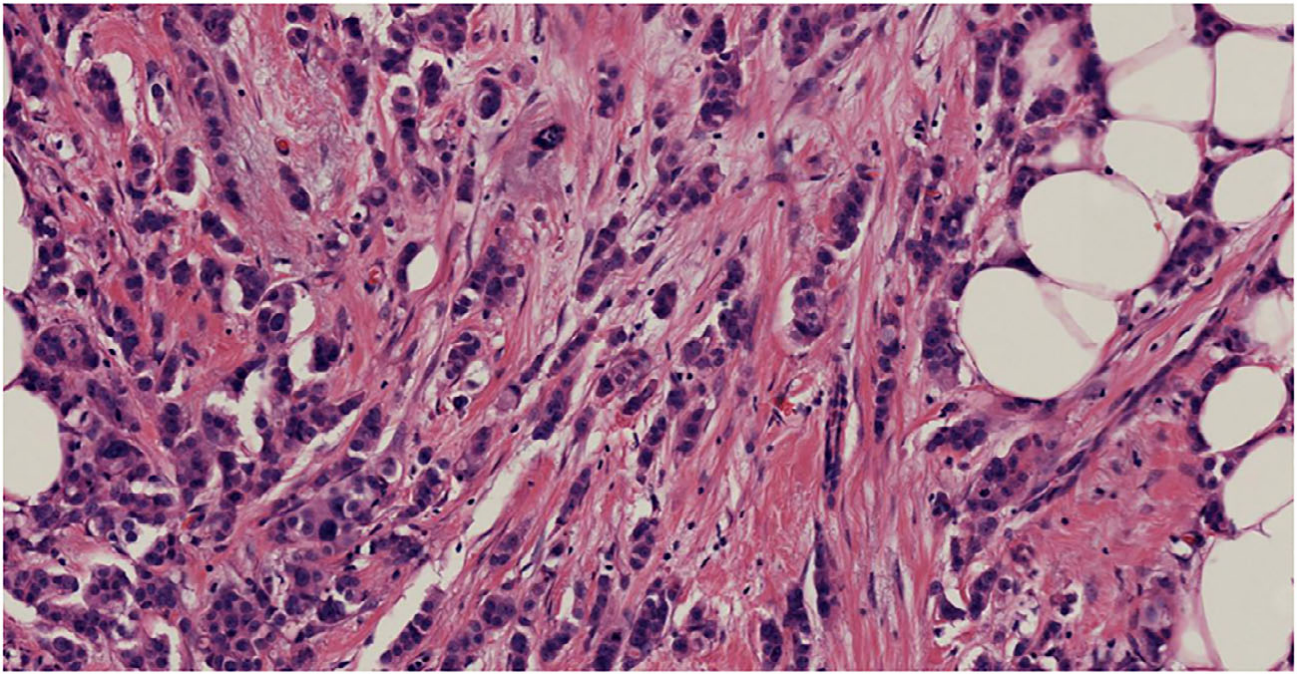
Pathologic findings of the left breast lesion. Microscopic examination shows an invasive breast carcinoma measuring 14 mm (stage III, hematoxylin and eosin stain, ×200)

**FIGURE 7:**
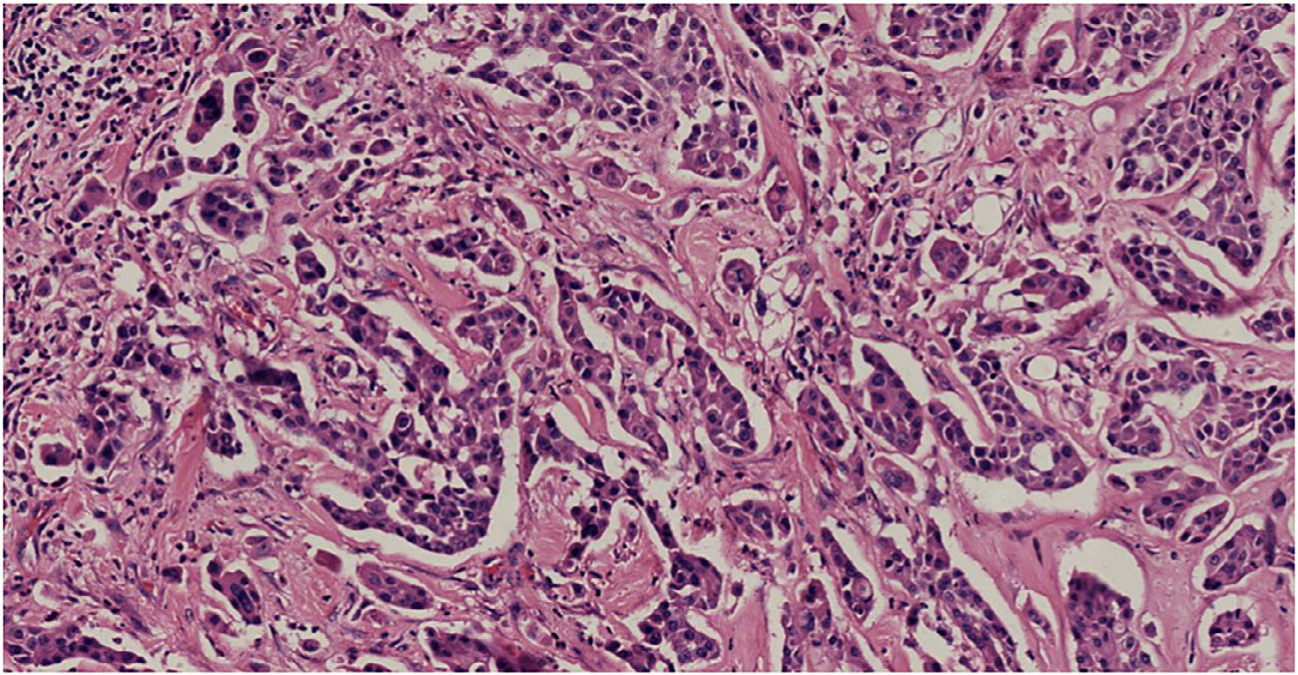
Pathologic findings of the right breast lesion. Microscopic examination shows an invasive breast carcinoma measuring 6 mm (stage II, hematoxylin and eosin stain, ×200)

US‐guided biopsy was performed on the two lesions, and histological examination results revealed both were invasive breast carcinoma (non‐special type, NST). The patient underwent double mastectomy, and intraoperative sentinel node biopsy was negative. Surgical specimens of the lesions were stained for pathological confirmation. The lesion in the left breast was invasive breast carcinoma (NST) measuring 14 mm (stage III, Figure 6) and the lesion in the right breast was also revealed to be invasive breast carcinoma (NST) measuring 6 mm (stage II, Figure 7).

## 3 DISCUSSION

The patient had BBC with one palpable tumor located in the marginal area of the upper inner quadrant of the left breast and one non‐palpable tumor located in the marginal area of the upper inner quadrant of the right breast. As described, AI‐breast US successfully located the two tumors, while automated breast ultrasound scanning, HHUS scanning, mammography (MG) and MRI examination all failed to detect the tumor in the right breast.

By MRI examination, no area with an enhancement pattern for malignancy was observed in the right breast, therefore no tumor in the right breast was reported. The macro‐calcifications and untypical blood flow of the tumor might lead to mis‐detection or mis‐diagnosis by MRI.^4^, ^5^ As reported, the diagnostic performance of the two‐view scan technique in automated breast ultrasound was similar to the results of three‐view scans in women with small breasts. In this case, the two‐view scan was chosen because the patient had a breast cup size of B. The medial coverage was considerated to be proper in AP view because there was absence of abrupt cutoff of the fibroglandular tissue at the margin of view and including the fatty tissue.^6^, ^7^ But the lesion of right breast was located in the fatty tissue far from the nipple, with a second reading of the scanning data by automated breast ultrasound, only part of the lesion in the right breast was shown in AP view. Women with dense breasts have been found to have a lower detection rate of breast cancer by MG due to the masking effect of dense tissue in Asian women. Some subtle MG findings or other lesions present in a marginal anatomic area have therefore been overlooked or misinterpreted.^8^, ^9^

The bilateral breast lesions were missed by HHUS screening at another hospital. The accuracy of breast screening is related to the radiologist's experience. In this case, the most important reason for missed diagnosis of the left breast lesion was the lack of understanding of a non‐mass lesion, which is defined as a discrete and identifiable area which shows altered echotexture compared with that of the surrounding breast tissue and does not conform to a mass shape. The failure to detect the tumor in the right breast by HHUS screening might be caused by atrophy of the patient's breast glands and the particular position of the tumor. The tumor was located in the marginal area of the breast and no obvious glandular tissue was observed surrounding it. In breast US scanning, the general radiologist usually takes the margins of glandular tissue as reference to identify the margins, leading to insufficient coverage. Another reason for the mis‐detection might be that the sonographers were more focused on evaluation of the palpable tumor in the left breast due to the relative low incidence of BBC and evaluation of the right breast was therefore lax. Standardized scanning reducing human errors or biases are important in breast US scanning.^10^, ^11^, ^12^

AI‐breast US^13^ is a standardized breast scanning technique based on conventional 2D US scanning and electromagnetic tracking and positioning technology by introducing a mattress electromagnetic field generator and integrating tracking coils in the transducer. AI‐breast US provides a calibrated GPS map of the scanned images in relation to the breast anatomy to document full coverage of the breast during the acquisition phase. In this case, for AI‐breast scanning, the technician utilized the up/down scanning leveraging experiences from automated breast ultrasound scanning. With the intuitive tool of AI‐breast to visualize the scanning procedure by brushing the screened areas green and displaying the result in a thumbnail of the breast anatomy, full coverage of the breast was ensured during scanning. By reviewing the scanning data without any blind areas, the two tumors were successfully detected. Compared with conventional HHUS, AI‐breast US firstly increases the diagnostic confidence of US doctors by reducing blind areas during scanning and also speeds up the workflow by the use of intuitive tools. Secondly, AI‐breast reduces dependence on the examiner's experience in breast US scanning by adopting standardized scanning technology which is easy for technicians to master. Thirdly, all of the breast US scanning data can be recorded in AI‐breast, including auto‐annotation of the lesion information, bookmarks of key frames within the loop, and quick access to the orthogonal plan from a reference point, which makes the longitudinal follow‐up of patients with breast lesions more convenient.

Cases of BBC are rare, and early diagnosis, which can significantly improve disease‐specific survival, depends on breast imaging. The most common method of screening for breast cancer in Asian women is US, but the performance of ultrasonologists varies to a large extent. Unlike HHUS, AI‐breast US uses a reproducible and less operator‐dependent process for image acquisition. In this case, AI‐breast US demonstrated its value in the detection of BBC, indicating its advantages in the diagnosis and follow‐up of patients with bilateral breast lesions. Meanwhile, with the trial to separate the screening procedure and detection or diagnosis procedure in this study, AI‐breast may initiate new workflows for breast US examination to improve coverage. Misdiagnosis and missed diagnosis are common in cases of BBC because of lack of attention to the contralateral breast after observation of a suspect lesion. Sonographers must not be satisfied with finding just one lesion, but must search carefully for others, whether ipsilateral or contralateral.

## Data Availability

All data and images generated or used during the study appear in the submitted article.
